# Recent Outbreaks, Resistance Trends, and Control Measures in *Candida auris* and *Candida glabrata* Infections

**DOI:** 10.3390/jof12060436

**Published:** 2026-06-15

**Authors:** Sepinoud Raeisi, Priya Madhavan, Diajeng Sekar Adisuri

**Affiliations:** 1 School of Medicine, Faculty of Health and Medical Sciences, Taylor’s University, 1 Jalan Taylors, Subang Jaya 47500, Malaysia; sepinoudraeisi1@gmail.com (S.R.); sekar.adisuri@gmail.com (D.S.A.); 2 Digital Health and Medical Advancement Impact Lab, Taylor’s University, 1 Jalan Taylors, Subang Jaya 47500, Malaysia

**Keywords:** multidrug resistance, healthcare-associated outbreaks, clade diversity, genomic surveillance, antifungal stewardship

## Abstract

The global rise in multidrug-resistant (MDR) fungal pathogens has positioned *Candida auris* and *Candida glabrata* as major threats to public health. In recent years, these pathogens have increasingly been reported beyond traditional hospital settings, including neonatal intensive care units, long-term care facilities, oncology wards, and post-pandemic critical care environments. International surveillance bodies, including the Centers for Disease Control and Prevention (CDC), European Centre for Disease Prevention and Control (ECDC), World Health Organization (WHO), and regional monitoring networks, have documented escalating antifungal resistance, complex outbreak dynamics, and persistent gaps in infection control implementation. *C. auris* has emerged as a major etiological agent of healthcare-associated outbreaks, particularly in intensive care and neonatal units. Surveillance data indicate that a high proportion of *C. auris* isolates exhibit resistance to azoles, often exceeding 80% in some regions, while echinocandin resistance remains variable. Resistance patterns have evolved from predominantly azole resistance to broader multidrug-resistant phenotypes, including treatment-emergent echinocandin resistance. Six genetically distinct clades (I–VI) have been identified, with Clades I, III, and IV associated with large-scale outbreaks, whereas available data suggests that Clades II, V, and VI are more geographically restricted, although evidence for the recently described clades remains limited. *C. glabrata* is increasingly recognized as a major cause of invasive candidiasis, with rising resistance reported across multiple regions. While reduced azole susceptibility was historically predominant, emerging evidence highlights rising dual azole–echinocandin resistance, adaptive microevolution during antifungal therapy, and biofilm-associated tolerance mechanisms. Despite these advances, significant gaps persist in global resistance surveillance and in the mechanistic understanding of virulence and antifungal adaptation. Current mitigation strategies include antifungal stewardship programs, expanded resistance testing, and strengthened surveillance systems. Advances in rapid diagnostic technologies such as matrix-assisted laser desorption ionization–time of flight (MALDI-TOF) mass spectrometry, polymerase chain reaction (PCR)-based assays, and genomic surveillance have improved pathogen identification and outbreak detection, although accessibility remains limited in resource-constrained settings. This review examines emerging epidemiological, genomic, and antifungal resistance trends in *C. auris* and *C. glabrata* and highlights key priorities for improving diagnosis, surveillance, stewardship, and management of multidrug-resistant *Candida* infections.

## 1. Introduction

Invasive candidiasis remains a major global cause of morbidity and mortality among critically ill and immunocompromised patients, with reported mortality rates of 30–60% despite antifungal therapy [1,2,3]. While earlier clinical attention was predominantly centered on *C. albicans*, the epidemiology of invasive candidiasis has progressively shifted toward non-*albicans Candida* species, which are characterized by greater antifungal resistance, increased persistence within healthcare environments, and higher diagnostic complexity [2,3]. Species such as *C. tropicalis* and *C. parapsilosis* exhibit variable susceptibility patterns and emerging resistance traits [3]. More recently, *C. auris* and *C. glabrata* have emerged as two of the most clinically significant fungal pathogens owing to their increasing global prevalence, persistence within healthcare environments, and reduced susceptibility to multiple classes of antifungal agents [3,4,5].

Since its first identification in Japan in 2009, *C. auris* has rapidly emerged as a major healthcare-associated pathogen and has been linked to outbreaks across Asia, Europe, and the Americas [4,5,6,7]. Notably, outbreaks have been reported across multiple countries, underscoring the organism’s capacity for rapid healthcare-associated dissemination in diverse clinical settings [6,7]. Whereas early outbreaks were largely confined to tertiary hospitals and intensive care units (ICUs), more recent transmission events increasingly involve neonatal intensive care units (NICUs), long-term care facilities, and other high-risk settings, indicating broader adaptation to diverse healthcare environments [6,7]. Its clinical success has been attributed to efficient skin colonization, adherence to abiotic surfaces, prolonged environmental survival, and reduced susceptibility to commonly used disinfectants [6,7,8]. Importantly, resistance patterns have evolved from predominantly azole resistance to multidrug-resistant phenotypes, including increasing echinocandin resistance and sporadic reports of pan-resistant isolates [6,9,10]. At the molecular level, azole resistance could be associated with regulatory pathways and efflux mechanisms, whereas echinocandin resistance is mainly linked to mutations in *FKS1* [8,10].

*C. glabrata*, once regarded as a relatively low-virulence opportunistic yeast, is now recognized as a leading cause of candidemia and invasive candidiasis, particularly among elderly, critically ill, and previously azole-exposed patients [2,3,11]. Unlike *C. auris*, which is strongly associated with healthcare transmission, *C. glabrata* infections often arise from endogenous gastrointestinal or mucosal colonization followed by host invasion under antimicrobial or antifungal selective pressure [11,12]. This trend is particularly evident in regions with extensive antifungal use, where selective pressure has accelerated the emergence of resistance. Historically, reduced fluconazole susceptibility represented the principal therapeutic concern; however, recent studies increasingly report dual azole–echinocandin resistance, adaptive microevolution during therapy, and persistence-associated tolerance mechanisms that are likely to contribute to recurrent infections [13,14,15]. Resistance to azoles is commonly mediated by transcriptional regulatory changes and efflux activity, whereas echinocandin resistance is most often associated with hotspot mutations in *FKS* genes and adaptive cell wall remodeling responses [14,15,16].

Although *C. auris* and *C. glabrata* share multidrug-resistant phenotypes, they differ substantially in transmission dynamics, ecological behavior, and evolutionary adaptation. *C. auris* primarily threatens healthcare systems through outbreak transmission and environmental persistence, whereas *C. glabrata* more commonly compromises treatment success through host-associated persistence and therapy-driven resistance. These distinctions have direct implications for infection prevention policies, empirical antifungal selection, and stewardship strategies. In parallel, diagnostic pathways have evolved considerably. Earlier diagnostic approaches relied primarily on conventional culture and biochemical identification systems, whereas contemporary strategies increasingly incorporate MALDI-TOF mass spectrometry and molecular assays to improve species identification and resistance surveillance [17,18,19]. Nevertheless, access to these technologies remains uneven in many resource-constrained settings [17].

Despite growing awareness of these pathogens, important gaps remain in understanding their evolving epidemiology, resistance trajectories, virulence determinants, and optimal management strategies [3,4,20]. These limitations may hinder the development of effective therapeutics, infection prevention programs, and integrated global surveillance systems [1,20]. Therefore, coordinated international research efforts are required [3,20]. This review synthesizes recent literature on emerging outbreaks, evolving resistance patterns, diagnostic advances, and key clinical challenges in *C. auris* and *C. glabrata*.

## 2. Methods

This article provides a narrative review of the literature concerning *C. auris* and *C. glabrata*, emphasizing recent outbreaks, trends in antifungal resistance, genetic and genomic traits, and infection control strategies. The review primarily focused on publications from 2020 to 2025, aligning with the manuscript’s scope, while selectively incorporating earlier seminal studies and public health guidance documents as necessary to provide critical epidemiological, clinical, or methodological context.

Relevant literature was identified through major biomedical bibliographic databases, including PubMed, Scopus, and Web of Science. Combinations of keywords such as “*C. auris*”, “*C. glabrata*”, “outbreak”, “epidemiology”, “antifungal resistance”, “azole resistance”, “echinocandin resistance”, “genomics”, “clade”, “infection control”, “surveillance”, “diagnostics”, and “antifungal stewardship” were used. The search strategy broadly followed combinations such as (“*C. auris*” OR “*C. glabrata*”) AND (outbreak OR epidemiology OR genomics) AND (“antifungal resistance” OR azole OR echinocandin). Additional relevant studies were identified through manual screening of the reference lists of eligible articles. Guidance documents and surveillance reports from major public health organizations, including the WHO, ECDC, and the CDC, were also reviewed when relevant to infection prevention, surveillance, and public health priorities.

English-language peer-reviewed original research articles, review papers, epidemiological and genomic studies, surveillance reports, and guideline or public health documents were considered if they were directly related to the scope of this review. Studies were prioritized based on their provision of recent or clinically relevant evidence regarding outbreaks, transmission dynamics, molecular resistance mechanisms, clade diversity, diagnostic advances, antifungal stewardship, or infection prevention and control strategies. Duplicate records, non-English publications, and sources not sufficiently relevant to the focus of the review were excluded. Approximately 120 publications were screened, from which 65 were selected based on relevance to the scope of this narrative review.

This review did not aim to be systematic and therefore did not follow PRISMA guidelines; instead, studies were selected to provide a representative and clinically relevant synthesis of current evidence. The focus was on synthesizing evidence from peer-reviewed studies, multicenter surveillance data, and official institutional guidance to provide a clinically relevant and up-to-date overview of the field.

## 3. *Candida auris*

### 3.1. Candida auris Clade Epidemiology and Outbreaks

**Figure 1:**
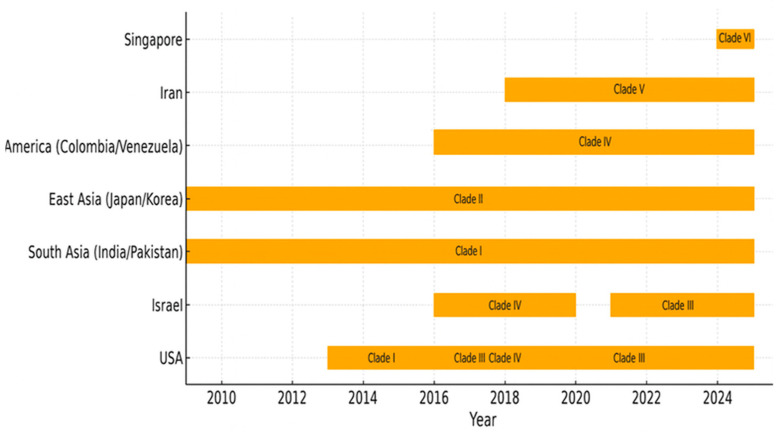
Timeline of *C. auris* clade dominance in different geographic regions (2009–2024). Timeline represents dominant reported clades based on published surveillance studies and is intended as a conceptual summary rather than a quantitative epidemiological analysis.

To date, six clades (I–VI) have been identified, each associated with its own distinct geographic distributions and phenotypic characteristics (Figure 1) [21,22].

First identified in 2009 in Japan, early isolates were predominantly associated with Clade II (East Asian clade), which exhibits lower thermotolerance, reduced virulence, and a lower propensity for large-scale outbreaks compared with other lineages [21,23,24]. However, genomic evidence suggests that *C. auris* did not arise from a single origin. Instead, it may have emerged independently across multiple geographic regions as genetically distinct clades under environmental and antifungal selective pressures [22,24,25]. In contrast to *C. glabrata*, which is primarily associated with endogenous infection, *C. auris* may demonstrate significant potential for environmental persistence and healthcare-associated transmission.

Clades I (South Asian), III (African), and IV (South American) are responsible for most of the global healthcare-associated outbreaks, whereas Clade II remains largely confined to East Asia and is less frequently associated with invasive outbreaks [5,7,24]. The recently described Clade VI, also referred to as the Singapore clade, appears geographically restricted and remains incompletely characterized [21,23]. These clade-specific differences, including variations in thermotolerance, aggregation behavior, environmental persistence, and antifungal susceptibility, are likely to play a critical role in shaping transmission dynamics and outbreak potential [8,26].

Over the past decade, *C. auris* has transitioned from an emerging pathogen to a major cause of healthcare-associated outbreaks worldwide. This shift is most likely driven by the increasing dominance of highly transmissible clades, particularly Clades I, III, and IV, which exhibit enhanced environmental resilience and the ability to persist on abiotic surfaces [5,8,9]. Combined with reduced susceptibility to commonly used antifungal agents, these characteristics could facilitate sustained transmission in high-risk healthcare environments [5,9].

Geographically, the global spread of *C. auris* may reflect both clonal expansion and repeated introductions of distinct clades into healthcare environments. In the United States, early cases were primarily associated with Clade I; however, subsequent genomic analyses demonstrated independent introductions of Clades III and IV, which now co-exist in healthcare settings, including long-term care facilities [22,25]. In some regions, Clade III has become dominant, possibly due to increased thermotolerance and enhanced persistence in hospital environments, facilitating transmission [8,26]. Similar shifts have been observed in Israel, where Clades I and III have increasingly replaced previously dominant lineages in intensive care settings [7].

In the Indian subcontinent, particularly in densely populated regions of India and Pakistan, Clade I remains the predominant lineage and is frequently associated with multidrug-resistant and occasionally pan-resistant isolates [24,26]. These strains continue to drive outbreaks in tertiary care hospitals, highlighting ongoing challenges in infection control and surveillance [7]. In South America, outbreaks in countries such as Colombia and Venezuela are mainly linked to Clade IV, with phylogenetic evidence suggesting local clonal expansion within healthcare systems [4,26].

Across healthcare settings, *C. auris* outbreaks are most frequently reported in intensive care units (ICUs), neonatal intensive care units (NICUs), and long-term care facilities, where factors such as high patient density, invasive procedures, and extensive antimicrobial use create favorable conditions for transmission [5,9]. The pathogen’s ability to persist on environmental surfaces and its reduced susceptibility to commonly used disinfectants, including quaternary ammonium compounds and chlorhexidine, further complicate infection control efforts [6,9].

At the molecular level, the increasing prevalence of multidrug-resistant and, in some instances, pan-resistant *C. auris* isolates has raised substantial clinical concern. The emergence of resistance during antifungal therapy, coupled with clade-specific susceptibility differences, highlights the role of antifungal selective pressure in shaping both resistance evolution and clade dominance [22,24,25].

Overall, *C. auris* demonstrates remarkable adaptability and phenotypic plasticity. The co-circulation of multiple clades within healthcare systems may reflect parallel evolutionary trajectories. These patterns are likely driven by environmental stressors and antifungal selective pressures [7,19]. In some regions, clade replacement events have been observed, whereby newly introduced, more thermotolerant or persistent lineages displace previously established strains, indicating ongoing evolutionary selection within clinical environments [22,24,25].

Given the persistence of *C. auris* and its ability to exploit weaknesses in healthcare systems, there is a critical need for integrated global surveillance frameworks capable of facilitating real-time data sharing. Coordinated genomic monitoring, clade tracking, and rapid molecular diagnostics are essential for controlling transmission and preventing large-scale outbreaks [4,7,19].

### 3.2. Genetic Diversity and Clade Characteristics

**Figure 2:**
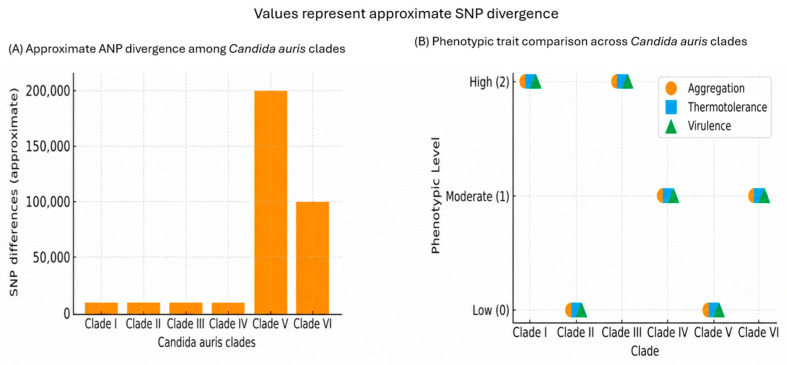
Single-nucleotide polymorphism (SNP) divergence (**A**) and phenotypic trait comparison (**B**) across *Candida auris* clades. Values are schematic approximations based on published genomic analyses [20,21,22].

As a haploid yeast with a compact genome of approximately 12–13 Mb distributed across seven chromosomes, *C. auris* exhibits substantial genomic plasticity, which may contribute to its adaptive capacity [21]. Comparative genomic analyses based on whole-genome sequencing (WGS) and related molecular approaches suggest that these clades are highly divergent, differing by tens of thousands of single-nucleotide polymorphisms (SNPs) (Figure 2). Current genomic evidence suggests that these clades may have evolved through parallel divergence from a common ancestral population under region-specific environmental and antifungal selective pressures [20,21,22].

While the six proposed clades (I–VI) of *C. auris* represent distinct evolutionary lineages within the species [21,22], collectively they still retain conserved core biological pathways, including ergosterol biosynthesis, stress response systems, and efflux-mediated transport mechanisms.

Clade I is the most widely distributed lineage and has been most frequently associated with large-scale healthcare-associated outbreaks across multiple continents [7,21]. Clade III has also contributed to nosocomial transmission in several regions, and some studies suggest that its genomic background may be associated with traits such as biofilm formation and environmental persistence [6,8]. Clade IV is primarily associated with regional outbreaks in parts of South America and the Caribbean and appears less globally disseminated than Clades I and III, while still retaining clinically relevant virulence and stress tolerance traits [4,20].

Clade II has been more commonly associated with sporadic cases rather than widespread outbreaks [5,21]. Available evidence suggests lower thermotolerance and reduced aggregation capacity compared with more globally distributed clades, which may have contributed to its limited outbreak potential [5]. Clade V has been reported in only a limited number of clinical isolates and remains uncommon in global surveillance datasets [22]. Therefore, its apparent genomic divergence, thermotolerance profile, and limited epidemiological spread should be interpreted cautiously until larger datasets become available. The most recently described Clade VI, identified in Southeast Asia, represents a newly recognized lineage that is still under investigation [20,22]. Although its genomic distinctiveness suggests ongoing diversification within the species, its transmissibility, resistance profile, and clinical significance remain insufficiently defined.

Importantly, genetic variation in *C. auris* is not restricted to differences between clades; substantial heterogeneity also exists within individual clades. This intra-clade diversity is hypothesized to have arisen from point mutations, copy-number variations, and regulatory changes affecting genes involved in antifungal resistance and stress adaptation. Mutations in key loci such as *ERG11* and *FKS1* are strongly associated with resistance to azole and echinocandin antifungal agents, respectively [27,28]. Alterations in transcriptional regulators such as *UPC2* could increase the expression of resistance-associated genes, including efflux pumps and ergosterol biosynthesis enzymes. These changes are more likely to enhance antifungal tolerance [28,29]. Importantly, regulators such as *UPC2* may function as global amplifiers of resistance by upregulating ergosterol biosynthesis pathways [23,25,29]. These molecular mechanisms provide a possible link between genomic variation and clinically relevant phenotypes, including drug resistance, biofilm formation, and environmental persistence; however, these relationships are not always consistent across clades or clinical settings.

Epidemiological evidence suggests that clade distribution is influenced by both geographic expansion and repeated independent introductions into healthcare systems [20,23]. In some regions, multiple clades may co-circulate within the same healthcare facilities, reflecting global connectivity and patient transfer networks [20]. Over time, shifts in dominant clades have been observed in certain settings, potentially reflecting differences in environmental fitness, transmissibility, and antifungal selection pressure [21,23]. Such dynamics may reflect ongoing changes in local epidemiology and complicate infection prevention strategies.

However, clade-specific interpretations remain limited by uneven genomic surveillance and sampling bias. Most available WGS datasets are derived from outbreak investigations or reference laboratories, while isolates from low- and middle-income countries remain underrepresented. As a result, current associations between specific clades and phenotypes such as transmissibility, thermotolerance, biofilm formation, or resistance may partly reflect where sequencing has been performed rather than true global biological patterns. This limitation is particularly important for Clades V and VI, for which available isolate numbers remain small. Therefore, conclusions regarding clade-specific fitness, resistance, and clinical behavior should be considered provisional.

The genetic and phenotypic diversity observed within *C. auris* clades likely plays a central role in determining clinical behavior, transmissibility, environmental persistence, and resistance evolution [21,22]. However, in many low- and middle-income countries, genomic surveillance capacity remains limited, resulting in under-detection of emerging lineages and incomplete understanding of global clade distribution [19]. Advances in diagnostic technologies, including MALDI-TOF mass spectrometry, PCR-based assays, and whole-genome sequencing, have significantly improved species-level identification and clade differentiation compared with traditional biochemical methods [18,19,26]. Continued expansion of genomic surveillance is essential for accurately tracking transmission patterns, guiding infection control strategies, and supporting evidence-based antifungal therapy.

### 3.3. Drug Resistance Profiles in C. auris

Over time, the clinical importance of *Candida auris* has increased markedly due to its broad-spectrum antifungal resistance [4,6]. Antifungal resistance is not uniformly distributed across the six major phylogenetic clades (I–VI), with Clades I–IV most frequently associated with hospital outbreaks, whereas Clades V and VI remain geographically restricted and less well characterized (Table 1) [21,22,25]. Importantly, the resistance profile of *C. auris* has evolved from predominantly azole resistance to MDR and, in some cases, pan-resistant phenotypes involving resistance to azoles, echinocandins, and polyenes [6,24,30]. However, interpretation of clade-specific resistance patterns should remain cautious because available resistance data are unevenly distributed across regions and clades, and recently described lineages are represented by relatively small numbers of isolates.

Clade I is among the most resistant lineages and has been frequently associated with MDR and pan-resistant isolates [21,25]. Antifungal resistance within this clade is strongly associated with mutations in the *ERG11* gene, particularly substitutions such as Y132F, which reduce azole binding affinity [27,29]. In addition, overexpression of efflux pumps, such as *CDR1*, and transcriptional regulators such as *TAC1b* and *UPC2* enhances drug efflux and upregulates ergosterol biosynthesis pathways, further reducing intracellular drug accumulation and reinforcing resistance [25,27,29]. Nevertheless, these mechanisms do not always predict resistance phenotypes uniformly across isolates, suggesting that clade background, regulatory variation, gene dosage, and local antifungal exposure could modify the final susceptibility profile.

Echinocandin resistance is primarily mediated by mutations in the *FKS1* gene, particularly within hotspot regions, which alter the structure of β-1,3-D-glucan synthase and reduce drug-binding affinity [28,31]. Such mutations may emerge during antifungal therapy and have been associated with clinical treatment failure [10,28]. Amphotericin B resistance has also been reported, although its molecular basis remains poorly characterized [25]. Unlike azole and echinocandin resistance, which are more consistently associated with *ERG11* and *FKS1* alterations, respectively, amphotericin B resistance has not yet been linked to a single reproducible genetic mechanism. Proposed explanations include alterations in sterol composition, membrane remodeling, and stress-response pathways, but these associations vary between studies and require further functional validation. Flucytosine resistance has been associated with mutations in genes such as *FUR1* and *FCY2*, which impair drug activation in fungal cells [24].

Clade III, originally identified in Africa and later reported in multiple regions including the United States, shares several resistance features with Clade I [21,25]. Reduced susceptibility to azoles is commonly associated with *ERG11* mutations or gene amplification [25]. However, resistance to echinocandins and amphotericin B appears less frequent compared with Clade I [25]. This apparent difference may reflect true biological variation between clades, but it may also be influenced by differences in sampling density, antifungal exposure history, and regional surveillance capacity.

Clade II generally exhibits lower resistance levels [21,22]. Fluconazole resistance has been reported and is frequently associated with mutations in transcriptional regulators such as *TAC1b*, with comparatively limited efflux activity and rare echinocandin resistance [27]. This clade is also characterized by lower virulence and outbreak potential [22]. However, because Clade II is less commonly associated with large-scale outbreaks, its resistance profile may be less comprehensively characterized than those of more widely disseminated clades.

Clade IV is likely to demonstrate moderate resistance patterns, with reported *ERG11* mutations contributing to azole resistance and occasional *FKS1* variants associated with reduced echinocandin susceptibility [25,28]. In contrast, Clade V remains poorly characterized but appears to retain relatively higher susceptibility to antifungal agents and has not been strongly linked to large-scale outbreaks [22]. Given the limited number of available Clade V isolates, these observations should be considered preliminary rather than definitive.

The recently described Clade VI represents a genetically distinct lineage with substantial divergence from other clades [16]. Although reduced susceptibility to azoles and echinocandins has been observed in certain isolates, the overall resistance profile and clinical significance of this clade remain poorly characterized [16]. At present, the limited availability of phenotypic and genomic data prevents firm conclusions regarding whether Clade VI has a distinct resistance trajectory or whether reported susceptibility patterns reflect isolate-level variation.

At a mechanistic level, pan-resistance in *C. auris* is thought to arise through the cumulative interaction of multiple pathways rather than a single mutation. Rather than representing a uniform phenotype, pan-resistance likely develops through different combinations of mechanisms across isolates and treatment contexts. These include *ERG11* mutations, increased efflux pump activity, transcriptional rewiring involving *TAC1b*, *MRR1*, and *UPC2*, and eventual *FKS1* mutations [25,27,30]. However, the relative contribution of each pathway to clinical treatment failure remains incompletely resolved, particularly because many mechanistic associations are derived from limited isolate collections, in vitro studies, or outbreak-specific datasets.

Epidemiological evidence suggests that the evolution of antifungal resistance in *C. auris* has accelerated in recent years, particularly in high-burden healthcare settings [6,9]. Increasing reports of MDR and pan-resistant isolates suggest a dynamic evolutionary process driven by antifungal use and infection control challenges [6,9]. Nevertheless, the apparent acceleration of resistance can also be partly influenced by improved detection, expanded surveillance, and increased reporting from outbreak settings. Therefore, resistance trends should be interpreted alongside changes in diagnostic capacity and surveillance intensity.

Despite advances in understanding resistance mechanisms, important gaps remain. The molecular mechanisms underlying amphotericin B resistance remain incompletely elucidated [25]. In addition, the genotype–phenotype relationship in *C. auris* resistance is not always straightforward, as identical or similar resistance-associated mutations may not produce equivalent susceptibility patterns across different clades or clinical backgrounds. Continued genomic surveillance and the development of novel antifungal agents will be critical to address the expanding threat of antifungal resistance in *C. auris* [20,32].

**Table 1:** Summary of molecular mechanisms, effect and clinical impact of antifungal resistance in *C. auris* across clades.

Drug Class/Resistance Type	Molecular Mechanism/Genes Involved	Effect	Clinical Impact	Refs
Azole resistance	*ERG11* mutations *(e.g., Y132F, K143R)*; overexpression of *CDR1*, *TAC1b* and *UPC2*	Reduces azole binding affinity, enhances drug efflux, decreases intracellular drug accumulation.	Outbreak-prone, persistent environmental contamination, linked to pan-resistant cases.	[21,25,27,30,33]
Amphotericin B resistance	Putative alterations in ergosterol biosynthesis pathways, including *ERG6*-associated mechanisms	Altered membrane sterol composition resulting in reduced amphotericin B binding	Linked to pan-resistant cases.	[33]
Echinocandin resistance	Mutations in the *FKS1* gene	Alters the structure of β-1,3-D-glucan synthase and reduces drug-binding affinity	Associated with treatment failure and reduced efficacy of first-line therapy	[7,25,29]
Flucytosine resistance	Mutations in genes *FUR1* and *FCY2*	Impairs drug activation in fungal cells	Associated with clinical treatment failure	[24]

### 3.4. Infection Control Against C. auris

Given its ability to spread within healthcare systems and persist in the environment, *C. auris* requires stringent, multi-layered infection control measures. Early detection and screening are essential to limit transmission. Routine skin screening of high-risk patients and point-prevalence surveys in outbreak-prone settings can help identify colonized individuals and enable timely intervention [8,19,26]. Increasing recognition of asymptomatic colonization has shifted infection control strategies from reactive approaches toward proactive, surveillance-based models [7,19].

Isolation and contact precautions should be implemented immediately after detection. For colonized or infected patients, single-room accommodation, strict hand hygiene, and appropriate personal protective equipment are recommended to reduce healthcare-associated transmission [8,19]. The ability of *C. auris* to colonize skin and persist on surfaces further reinforces the need for strict adherence to these measures, particularly in intensive care units and long-term care facilities [7,19,22]. Compared with other *Candida* species, *C. auris* does demonstrate enhanced environmental persistence and transmission efficiency [6,7].

Environmental cleaning and disinfection are critical components of outbreak control. Standard disinfectants show reduced efficacy against *C. auris*, particularly in the presence of biofilms [19,22]. This reduced efficacy is partly attributed to biofilm formation and tolerance to commonly used disinfectants [7,19]. As a result, enhanced disinfection strategies, including hydrogen peroxide vapor and ultraviolet-C irradiation, are recommended in high-risk settings [8,19,26]. These approaches address the limitations of conventional cleaning protocols and improve decontamination outcomes [8,26].

Institutional infection control strategies should also include environmental monitoring and improved inter-facility communication. Patient transfers without proper notification have contributed to the introduction of *C. auris* into previously unaffected regions [7,19,26]. Coordinated communication between healthcare facilities is therefore essential to limit cross-institutional spread [7,26].

Rapid diagnostic methods play a key role in early detection and containment. Techniques such as polymerase chain reaction, MALDI-TOF mass spectrometry, and other molecular assays enable accurate identification of *C. auris* and support timely implementation of control measures [17,18]. These methods represent a major improvement over conventional biochemical identification, which frequently resulted in misidentification [17,18]. However, limited access to advanced diagnostics in resource-limited settings remains a critical barrier to effective outbreak control [17].

At a population level, genomic surveillance and antifungal stewardship are also essential for long-term outbreak management. Whole-genome sequencing has been used to track transmission pathways, identify clade distribution, and detect emerging resistance patterns [7,21,25]. Integration of genomic data into national surveillance systems can improve outbreak detection and response [7,21]. In addition, antifungal stewardship programs help reduce selective pressure and limit the emergence of resistant strains [3,6].

In summary, control of *C. auris* requires a coordinated approach that includes early screening, strict isolation precautions, effective environmental disinfection, rapid diagnostics, and genomic surveillance. The transition from reactive containment to proactive surveillance-based strategies reflects improved understanding of the organism’s transmission and persistence. Sustained international collaboration and improved access to diagnostic and surveillance tools are essential to effectively contain its ongoing global spread [3,6,7].

## 4. *Candida glabrata*

### 4.1. Candida glabrata Outbreaks

Unlike *C. auris*, which emerged rapidly as a globally recognized healthcare-associated pathogen following large-scale hospital outbreaks, *C. glabrata* has followed a more gradual epidemiological trajectory. Historically regarded as a low-virulence commensal of the gastrointestinal and genitourinary tracts, *C. glabrata* has progressively transitioned into a clinically significant opportunistic pathogen over recent decades [2]. This shift has occurred alongside the expansion of elderly and immunocompromised populations, increased use of invasive medical procedures, and widespread exposure to broad-spectrum antibiotics and azole antifungals [1,34]. In contrast, the emergence of *C. auris* has been implicated in nosocomial transmission, environmental persistence, and large institutional outbreaks [6]. Thus, while *C. auris* is likely to represent an outbreak-driven model of emergence, *C. glabrata* reflects a host- and treatment-associated model of increasing prevalence. Unlike *C. auris*, which transmits efficiently in healthcare environments, *C. glabrata* infections primarily emerge through host-associated and treatment-driven mechanisms.

The epidemiological expansion of *C. glabrata* has therefore been less abrupt but more sustained than that of *C. auris*. Rather than causing widespread epidemics, *C. glabrata* is primarily associated with sporadic clusters or confined hospital transmission events [12]. Nonetheless, it remains a major clinical concern due to the increasing incidence of invasive candidiasis and the rapid rise in MDR [3]. Surveillance data from the United States and Europe indicate that *C. glabrata* accounts for approximately 20–25% of candidemia cases, predominantly affecting elderly and immunocompromised patients [2,3,35]. These trends demonstrate the transition of *C. glabrata* from a low-virulence commensal organism to a clinically important opportunistic pathogen.

A key distinction between the two species lies in their transmission dynamics. Most *C. glabrata* infections are endogenous and typically originate from colonization of the gastrointestinal and genitourinary tracts, particularly in patients with prior azole exposure [12]. In contrast, transmission of *C. auris* is associated with contaminated healthcare environments, patient-to-patient transmission, and prolonged survival on abiotic surfaces [7]. The persistence of *C. glabrata* in intensive care, oncology, and transplant settings has been attributed to persistent reduced azole susceptibility and the rapid development of acquired resistance during antifungal therapy [11]. Importantly, significant environmental shedding of *C. glabrata* has not been conclusively demonstrated. Instead, prolonged antifungal exposure, particularly during hospitalization, exerts dominant selective pressure driving resistance emergence and persistence [15]. These findings indicate that the increasing prevalence of *C. glabrata* is tightly linked to antifungal pressure, host vulnerability, and healthcare-associated selection rather than environmental persistence alone, underscoring the critical importance of antifungal stewardship [1].

In Asia, the epidemiology of *C. glabrata* has evolved alongside increasing antifungal use and the growing population of high-risk hospitalized patients. In Japan and South Korea, *C. glabrata* accounts for approximately 15–20% of candidemia cases, reflecting patterns like those observed globally [2]. During the same period, *C. auris* has raised significant concern because of its ability to spread rapidly within healthcare environments [6], whereas *C. glabrata* remains more strongly associated with endogenous infection and treatment-driven resistance. Notably, the increasing incidence of echinocandin resistance among patients receiving prolonged antifungal therapy, particularly those with hematological malignancies, remains a major clinical concern [16]. Moreover, the lack of reporting combined with the absence of comprehensive genomic surveillance has limited the scope of outbreak investigations in Southeast Asia. This represents a major limitation in low- and middle-income countries (LMICs), where limited routine genomic sequencing is likely to obscure the true prevalence and dissemination of resistant *C. glabrata* strains [2].

Although *C. glabrata* does not exhibit the same degree of environmental persistence or rapid cross-transmission as *C. auris*, its evolving resistance trajectory suggests progressive adaptation to healthcare environments. The emergence of echinocandin-resistant isolates associated with *FKS1* and *FKS2* mutations, including reports of clonal mutant strains, could suggest a tendency toward clonal adaptation under antifungal selective pressure [16]. At the same time, evolutionary genomic studies suggest that resistance in *C. glabrata* is predominantly polyclonal and not strongly associated with specific lineages [13]. However, resistance-conferring mutations to azoles and echinocandins can facilitate the secondary spread of resistant strains within healthcare settings [36]. Therefore, whereas *C. auris* spreads predominantly through environmental persistence and high transmissibility, *C. glabrata* spreads primarily through antifungal selection pressure, endogenous reservoirs, and subsequent dissemination of resistant strains. The evolution of resistant *C. glabrata* populations therefore warrants vigilant and continuous genomic surveillance to mitigate the risk of nosocomial transmission [3].

In summary, the epidemiological emergence of *C. glabrata* differs fundamentally from that of *C. auris* in both tempo and underlying mechanisms. Whereas *C. auris* has emerged through rapid outbreak-associated transmission [6], *C. glabrata* has increased in prevalence through host susceptibility, antifungal exposure, endogenous colonization, and acquired resistance [1,34]. Although *C. glabrata* remains less likely to cause large-scale outbreaks, its increasing prevalence, limited availability of rapid diagnostics, high mortality associated with bloodstream infections, and rising multidrug resistance represent significant and growing public health challenges [3]. Targeted genomic surveillance, rapid diagnostic implementation, and strict antifungal stewardship are therefore essential to prevent sporadic clusters from evolving into persistent hospital transmission events [1]. If left unaddressed, *C. glabrata* could progressively shift from a commensal organism to a major multidrug-resistant pathogen with clinical impact approaching that of *C. auris*.

### 4.2. Candida glabrata Genetics

*C. glabrata*, also known as *Nakaseomyces glabratus*, is classified as a haploid yeast and a member of the Saccharomycetaceae family. It is more closely related to *S. cerevisiae* than to *C. albicans* [12,37]. Its genome is approximately 12.3 megabases (Mb) in length, spanning 13 chromosomes, and closely resembles that of *S. cerevisiae*. However, most genes involved in hyphal formation are absent, including key regulatory and structural components required for filamentation in *C. albicans*, such as *EFG1*, *CPH1*, *HWP1*, and *ALS3*. As a result, *C. glabrata* does not form true hyphae, remains strictly yeast-form, and exhibits a monomorphic morphology, which may contribute to survival under antifungal pressure [12,34].

From a ploidy perspective, *C. glabrata* is predominantly haploid, whereas *C. albicans* is diploid. This allows genetic mutations in *C. glabrata* to be expressed directly at the phenotypic level, facilitating rapid adaptation to environmental and antifungal stress [12]. In contrast, *Candida auris* also does not produce true hyphae but exhibits well-defined phylogenetic clades that support global transmission tracking [21].

Unlike *C. auris*, which is structured into distinct cross-regional clades, *C. glabrata* displays greater within-species genetic diversity but lower phylogenetic resolution and lacks formal clade classification [13,36]. Consequently, antifungal resistance in this species is not clade-specific but is instead driven by sequence type (ST)-specific microevolution [13]. This distinction is important because the absence of a formal clade framework limits direct comparison with *C. auris* and makes it more difficult to assign resistance patterns to stable population-level lineages.

Despite this lower phylogenetic differentiation, *C. glabrata* populations can be effectively resolved using multilocus sequence typing (MLST) and WGS, which define region-specific STs such as ST3 and ST7 in Europe and North America and ST55 and ST63 in Asia [13]. Although these STs are not equivalent to clades, they function as epidemiological markers that enable tracking of transmission and geographic distribution, but their clinical significance remains incompletely defined because ST assignment does not consistently predict resistance phenotype, virulence potential, or treatment outcome.

The observed genetic diversity in *C. glabrata* is primarily attributed to microevolutionary processes, including the accumulation of single-nucleotide polymorphisms (SNPs), chromosomal rearrangements, and aneuploidy [13,34]. However, genomic surveillance of *C. glabrata* resistance remains underreported in low- and middle-income countries (LMICs), leading to potential bias in the representation of its global genetic and geographical diversity [3]. This under-sampling could obscure local transmission events, emerging resistant STs, or region-specific evolutionary trajectories.

At the molecular level, the adaptability of *C. glabrata* is mediated through multiple genetic mechanisms. Gain-of-function mutations in the transcription factor *PDR1* enhance the expression of drug efflux pumps, including *CgCDR1*, *CgCDR2*, *SNQ2*, and *PDH1*, thereby promoting rapid azole resistance under antifungal exposure [14,35]. However, *PDR1*-mediated resistance does not fully explain all resistant phenotypes, indicating that additional regulatory pathways, transporter activity, and host-associated selective pressures are more likely to contribute to resistance evolution. These mutations can also contribute to isolate-level differentiation in epidemiological studies.

In addition, members of the hexose transporter family (*CgHXT4/6/7*) may act as azole importers, and even in the absence of *PDR1*, reduced intracellular drug accumulation and increased resistance have been observed [34]. Furthermore, *PDR1*, together with the zinc-cluster transcription factors *HAP1A* and *HAP1B*, regulates *ERG* gene expression. Deletion of *HAP1B* results in reduced *ERG* expression and ergosterol levels, increasing susceptibility to azoles and linking oxygen sensing to sterol biosynthesis [38,39]. Together, these findings suggest that azole resistance in *C. glabrata* cannot be interpreted solely through efflux-mediated mechanisms, but also through sterol regulation, oxygen sensing, and metabolic state.

Structural adaptations also contribute to resistance. Overaccumulation of chitin, cell wall thickening, and associated mutations can confer resistance to echinocandin-induced lysis [36,40]. In addition, *C. glabrata* can form echinocandin-tolerant persister cells and exhibit survival within macrophages. In murine models, amphotericin B has been shown to eradicate these intracellular persisters, although the extent to which these experimental observations translate to human infection, relapse, or treatment failure remains uncertain and requires further clinical validation [40].

A further unresolved issue is whether resistant *C. glabrata* should be interpreted primarily as a product of within-host microevolution under antifungal pressure or as a potential contributor to localized healthcare-associated transmission. Current evidence supports strong therapy-driven adaptation, but reports of related resistant isolates in hospital settings suggest that transmission could also occur under certain epidemiological conditions. This remains an important controversy because it affects whether surveillance should focus mainly on patient-level resistance emergence or also on infection-control tracking of resistant lineages [3,13,36].

In summary, the genome of *C. glabrata* is highly adaptable and resilient, enabling rapid microevolution and the emergence of resistant strains [41]. However, the absence of a formal clade structure, incomplete genotype–phenotype correlation, limited surveillance in LMICs, and uncertainty regarding the balance between within-host evolution and healthcare-associated transmission complicate interpretation of its genomic epidemiology. Its ST-based population structure, strong stress-responsive genetic plasticity, and limited genomic surveillance, particularly in LMICs, underscore the need for continuous global genomic monitoring [3,13].

### 4.3. Drug Resistance Profiles in C. glabrata

The significant role of *C. glabrata* in disease is underscored by its intrinsic reduced susceptibility to azoles and the rapid emergence of multidrug resistance during antifungal treatment, particularly in immunocompromised patients [12,34]. In contrast to *C. albicans*, *C. glabrata* is a non-hyphal yeast that relies on stress tolerance, metabolic flexibility, and specialized regulatory mechanisms to survive antifungal pressure [12,37]. Gene set enrichment analyses further indicate enhanced metabolic adaptability and additional traits contributing to persistence [12,31]. However, these adaptive traits do not translate into a single predictable resistance pathway, as resistance phenotypes are likely to vary according to isolate background, host environment, and antifungal exposure history.

Resistance development in *C. glabrata* follows a progressive, stepwise trajectory. The initial shift typically occurs under azole exposure, where gain-of-function mutations in the transcription factor *PDR1* drive overexpression of drug efflux pumps, including *CgCDR1*, *CgCDR2*, *SNQ2*, and *PDH1*, reducing intracellular azole accumulation [13,35]. Clinical studies of fluconazole-resistant isolates have confirmed a high prevalence of nonsynonymous *PDR1* mutations, highlighting the central role of efflux-mediated resistance [12]. Nevertheless, the presence of *PDR1* mutations alone may not fully explain resistance heterogeneity, indicating that azole resistance should be interpreted as a network-driven phenotype rather than a single-gene event.

Beyond *PDR1*, resistance is further shaped by alterations in drug transport and regulatory networks. Members of the hexose transporter family (*CgHXT4/6/7*) have been implicated in azole uptake, and even in the absence of *PDR1* mutations, transporter-related changes can influence intracellular drug accumulation and resistance [13]. At the regulatory level, *PDR1* acts together with zinc-cluster transcription factors *HAP1A* and *HAP1B*, which modulate *ERG* gene expression under azole and hypoxic stress conditions [38]. Deletion of *HAP1B* reduces *ERG* expression and ergosterol levels, increasing azole susceptibility and linking oxygen sensing to sterol biosynthesis [38]. These findings support a broader interpretation in which azole susceptibility is shaped not only by efflux activity but also by drug uptake, sterol regulation, oxygen sensing, and metabolic state.

As antifungal pressure intensifies, particularly with echinocandin use, resistance evolves through target-site modifications. Mutations in *FKS1* and *FKS2*, encoding subunits of β-1,3-glucan synthase, reduce echinocandin binding and represent a major mechanism of resistance [39]. Resistant isolates frequently harbor hotspot mutations in these genes, underscoring their diagnostic significance [34]. In addition, gene conversion events between *FKS* loci further contribute to adaptive evolution under therapeutic pressure [15]. However, the relationship between *FKS* mutations and clinical outcome is not always straightforward, because treatment failure could be influenced by drug exposure, immune status, source control, biofilm formation, and the emergence of tolerant subpopulations [15,34,39].

Structural adaptations reinforce this resistance phenotype. Enhanced deposition of chitin and cell wall thickening have been documented in resistant isolates. Mutations in *CHS3*, *CHS3B*, and *KRE5* are associated with reinforcement of cell wall architecture and increased chitin accumulation, potentially counteracting echinocandin activity [36]. These isolates are likely to also exhibit increased tolerance to environmental stressors, such as sodium dodecyl sulfate [42]. Nevertheless, the extent to which cell-wall remodeling alone predicts clinical echinocandin failure remains uncertain, as most evidence links these changes to resistant phenotypes at the isolate or experimental level rather than to standardized clinical outcome measures.

In parallel, *C. glabrata* demonstrates significant biofilm-associated resistance. Biofilm formation is mediated by adhesin-encoding genes such as *EPA1*, *EPA6*, and *EPA7*, along with regulatory genes including *BCR1*, *ACE2*, and *YAK1* [41]. Efflux pumps *CgCDR1* and *CgCDR2* remain important resistance factors in biofilm states, even in strains lacking *PDR1* mutations [35]. Biofilm-associated cells exhibit reduced susceptibility to azoles and echinocandins. This reduced susceptibility is associated with limited drug penetration, metabolic quiescence, active efflux, and activation of stress-response pathways. This is particularly relevant in catheter-associated bloodstream infections and infections involving implanted medical devices [12]. However, biofilm-associated resistance is difficult to compare across studies because experimental models, biofilm maturity, readout methods, and antifungal exposure conditions are not always standardized. Therefore, the clinical contribution of individual biofilm mechanisms should be interpreted cautiously.

A further layer of resistance arises from phenotypic heterogeneity, particularly the formation of drug-tolerant persister cells. Within macrophages, *C. glabrata* cells may enter a non-dividing oxidative stress-adapted state. This transition may result in transient echinocandin tolerance [40]. These persister populations act as reservoirs for subsequent resistance development. In contrast, amphotericin B has been shown to effectively eradicate intracellular persister cells in experimental models [31]. Although this finding is mechanistically important, the extent to which macrophage-associated persisters drive relapse, breakthrough infection, or treatment failure in human disease remains incompletely established.

Importantly, resistance development in *C. glabrata* is not uniform across all isolates. Unlike *C. auris*, which often exhibits clade-associated resistance patterns [21], *C. glabrata* resistance is highly heterogeneous and depends on sequence type (ST)-specific microevolution and local selective pressures [13]. Different isolates could also display distinct combinations of efflux activation, regulatory adaptation, structural remodeling, and persistence phenotypes. This heterogeneity creates a major interpretive challenge, as similar resistance phenotypes could arise through different genetic or physiological routes.

In contrast, *C. auris* frequently exhibits pre-existing resistance linked to specific clades, facilitating its spread as a resistant pathogen [4,21]. Thus, while *C. auris* primarily spreads as pre-adapted resistant lineages, *C. glabrata* evolves resistance progressively within the host during antifungal therapy. However, this distinction should not be viewed as absolute, because resistant *C. glabrata* strains may be associated with localized transmission under specific hospital conditions, whereas *C. auris* can also acquire additional resistance during therapy.

In summary, resistance in *C. glabrata* is a multifactorial and dynamic process involving genetic mutations, regulatory reprogramming, structural adaptation, metabolic shifts, and host–pathogen interactions [12,14,31] (Table 2). Current evidence supports a model of therapy-driven resistance evolution, but important uncertainties remain regarding the relative contribution of each mechanism to clinical failure, the standardization of biofilm and tolerance testing, and the translation of experimental persister models to human infection. The emergence of resistance to both azole and echinocandin classes necessitates intensified genomic surveillance, implementation of combination therapies, and development of novel antifungal strategies targeting persister cells, ergosterol homeostasis, and efflux systems [3,31]. These findings highlight the need for integrated global monitoring and therapeutic approaches [3].

### 4.4. Infection Control Against C. glabrata

Control of infections caused by *C*. *glabrata* still requires coordinated infection control and antifungal management strategies [2,12]. In contrast to *C*. *auris*, *C*. *glabrata* infections are primarily endogenous and do not efficiently transmit between patients [2,12]; consequently, earlier control approaches focused mainly on host-related risk management rather than interruption of patient-to-patient transmission. However, with the increasing incidence of antifungal resistance and more complex treatment challenges, effective control has become increasingly dependent on early recognition of infection risk and timely, targeted clinical management [1,3].

Historically, diagnosis of *C*. *glabrata* infections relied primarily on blood culture, which, although considered the gold standard, is limited by slow yeast growth, delayed results, low sensitivity, and prolonged turnaround times, often leading to empiric therapy [3]. As resistance rates increased, particularly among high-risk patients, the need for faster and more accurate diagnostics became critical, driving a shift toward advanced diagnostic tools. More recently, the introduction of MALDI-TOF mass spectrometry has enabled rapid species identification and substantially reduced diagnostic delays [18]. Diagnostic strategies have also evolved from purely phenotypic methods toward resistance-informed approaches. Early detection of echinocandin resistance increasingly relies on screening for hotspot mutations in *FKS1* and *FKS2* [16,39]. Consequently, all bloodstream isolates should undergo antifungal susceptibility testing, particularly for echinocandins, given the known propensity of *C*. *glabrata* to develop resistance [34].

In terms of infection control, earlier practices were largely limited to standard precautions such as hand hygiene and basic management of invasive devices, reflecting the organism’s limited capacity for interpatient transmission. However, as resistant infections became more prevalent and the role of catheters in secondary bloodstream infections became more evident, the importance of these measures increased significantly. Currently, although *C*. *glabrata* remains less environmentally persistent than *C*. *auris*, standard infection-control measures, including strict hand hygiene and careful management of catheters and other invasive devices, are essential to prevent endogenous translocation and bloodstream infection [1,2].

Clinical management has similarly evolved from empiric therapy toward targeted treatment. Historically, azoles were widely used empirically; however, recognition of the intrinsic azole resistance of *C*. *glabrata* and its strong ability to develop multifactorial resistance during antifungal exposure has limited this approach. Consequently, antifungal stewardship programs now play a central role, emphasizing reduction in unnecessary empirical or prophylactic use of fluconazole and echinocandins [1]. Therapeutic strategies have shifted toward preemptive and targeted antifungal therapy guided by diagnostic findings and patient-specific risk factors, while dosing strategies increasingly incorporate pharmacokinetic and pharmacodynamic (PK/PD) principles to optimize efficacy and reduce toxicity [3], particularly in oncology and hematology patients who are highly susceptible to colonization by resistant strains [1].

Surveillance has also evolved from limited local reporting to structured, multi-level monitoring systems. Previously, resistance data were primarily derived from individual case reports; however, increasing resistance complexity has necessitated systematic surveillance at institutional, regional, and national levels. Gain-of-function mutations in *PDR1*, along with mutations in *FKS1* and *FKS2*, are now recognized as clinically relevant molecular markers of resistance [12,16,39]. Strengthening surveillance increasingly involves integrating genomic monitoring of these mutations to support clinical decision-making and guide treatment strategies [14,15]. In comparison, in *C. auris*, genomic surveillance additionally plays a major role in tracking clonal spread and controlling healthcare-associated outbreaks [4,21], whereas in *C. glabrata,* the primary focus is on monitoring intrinsic evolution within the host and emergence of post-treatment resistance.

In summary, management of *C. glabrata* has evolved from a reactive approach based on delayed diagnosis and empirical therapy to a proactive framework integrating rapid diagnostics, resistance-guided treatment, antifungal stewardship, and structured surveillance. This transition has been driven by increasing antifungal resistance, advances in diagnostic technologies, and expansion of high-risk patient populations, underscoring the need for coordinated strategies combining early detection, infection control, individualized therapy, and continuous resistance monitoring to minimize clinical impact and prevent emergence of resistant infections [1,3].

## 5. Comparative Analysis

A direct comparison between *C. auris* and *C. glabrata* highlights fundamentally distinct epidemiological strategies, resistance trajectories, and clinical challenges (Table 3). Among non-albicans *Candida* species, *C. auris* and *C. glabrata* are among the most clinically significant, as both can cause invasive candidiasis with substantial morbidity and mortality; however, the observed differences in resistance patterns, transmission dynamics, and clinical behavior reflect distinct evolutionary and ecological strategies rather than isolated traits [1,12]. Therefore, the comparison between these species should focus not only on their individual mechanisms of resistance, but also on how these mechanisms shape different clinical and public health priorities.

A key divergence emerges in how resistance develops. In *C. auris*, fluconazole resistance is largely driven by stable genetic alterations such as *ERG11* mutations and constitutive overexpression of efflux pumps (*CDR1*, *MDR1*) [4,24,29]. This suggests that resistance is often pre-established at the population level, consistent with its clade-based genomic structure [21]. In contrast, *C. glabrata* typically acquires azole resistance during antifungal therapy, primarily through gain-of-function mutations in *PDR1*, leading to inducible overexpression of efflux pumps [12,13,35]. The implication is that resistance in *C. auris* is frequently a surveillance and containment problem, whereas resistance in *C. glabrata* is often a treatment-monitoring problem that emerges during patient management.

This distinction becomes more evident when examining echinocandin resistance. In *C. auris*, resistance remains relatively low (<5%) and is mainly associated with *FKS1* hotspot mutations, although localized increases have been reported [4,25,28]. In contrast, *C. glabrata* exhibits a broader and more variable range of echinocandin resistance, predominantly linked to mutations in *FKS2* and, to a lesser extent, *FKS1* [16,39]. This variability is associated with the capacity of *C. glabrata* to have host evolutionary diversification. Antifungal exposure may promote stepwise accumulation of resistance mechanisms during therapy. Clinically, this means that a single baseline susceptibility result could be less informative for *C. glabrata* when prolonged antifungal exposure, persistent infection, or inadequate source control allows resistance to evolve during treatment. The emergence of cross-class resistance further supports the involvement of adaptive stress-response pathways rather than fixed resistant lineages [31,34].

Differences in genomic architecture further reinforce these contrasting strategies. *C. auris* is structured into distinct geographic clades with defined antifungal susceptibility profiles [21,22], enabling clonal expansion and healthcare-associated transmission. In contrast, *C. glabrata* lacks sharply defined clades and instead exhibits high genetic plasticity driven by microevolution and sequence-type variation [12,13]. Thus, genomic data serves different practical purposes in the two species: for *C. auris*, they are central to outbreak reconstruction and clade tracking, whereas for *C. glabrata*, they are more useful for detecting within-host evolution, emerging resistance, or possible localized transmission events.

These genomic differences are hypothesized to translate directly into distinct epidemiological behaviors. The environmental persistence of *C. auris*, including survival on dry surfaces and reduced susceptibility to disinfectants, supports its role in healthcare-associated outbreaks [7,8,19]. In contrast, *C. glabrata* is primarily an endogenous commensal organism that causes infection following mucosal translocation, with limited survival outside the host and inefficient person-to-person transmission [2,12]. Accordingly, infection-control priorities differ: *C. auris* requires rapid screening, isolation, environmental decontamination, and inter-facility communication, whereas *C. glabrata* control depends more heavily on antifungal stewardship, catheter/source control, and early detection of resistance during therapy. In addition, insufficient genomic surveillance in LMICs may obscure the true extent of resistance evolution in *C. glabrata* [1].

Biofilm formation represents another mechanistic point of divergence. While both species form biofilms that reduce antifungal susceptibility, *C. auris* biofilms tend to be dense and matrix-rich, supporting environmental persistence and surface colonization [4,7]. In contrast, *C. glabrata* biofilms are more closely associated with metabolic activity, efflux-mediated resistance, and adaptive stress responses [12,41], consistent with its dynamic and treatment-associated resistance profile. This distinction is clinically relevant because biofilm control in *C. auris* is closely linked to environmental cleaning and outbreak prevention, whereas in *C. glabrata* it is more closely linked to device management and prevention of persistent or recurrent infection.

Despite these mechanistic differences, clinical outcomes are comparable. Bloodstream infections caused by *C. auris* show mortality rates of approximately 30–60%, which are broadly similar to those observed in *C. glabrata* candidemia (30–50%) [1,3]. However, the underlying drivers differ in *C. auris*, poor outcomes are often linked to delayed recognition, outbreak dynamics, environmental persistence, and limited infection-control capacity, whereas in *C. glabrata*, they are more strongly associated with host comorbidity, delayed effective therapy, source control, and resistance emergence during treatment.

Importantly, this comparison should not be interpreted as a rigid binary model. Although *C. auris* is typically described as a clonal outbreak pathogen and *C. glabrata* as a within-host adaptive pathogen, both species can show overlapping behaviors under certain conditions. *C. auris* may acquire additional resistance during therapy, while resistant *C. glabrata* strains could be associated with localized healthcare-associated clusters. Therefore, the distinction between the two species is best understood as a difference in dominant evolutionary and epidemiological tendencies rather than as an absolute separation [10,13,36].

Collectively, these findings indicate that antifungal resistance in *Candida* species cannot be explained by a single evolutionary framework. Taken together, the contrasting evolutionary and epidemiological trajectories of *C. auris* and *C. glabrata* highlight the need for species-specific surveillance and management strategies. While *C. auris* primarily represents a healthcare-associated transmission and containment challenge, *C. glabrata* poses a treatment and resistance-monitoring challenge driven by within-host adaptation. Future efforts should integrate genomic surveillance, antifungal stewardship, and rapid diagnostics to address the growing burden of multidrug-resistant *Candida* infections.

## 6. Future Directions

Despite substantial advances, significant gaps remain in understanding and controlling *C. auris* and *C. glabrata*, particularly in linking molecular resistance mechanisms with clinical and epidemiological outcomes [1,12]. A major priority is the development of integrated frameworks that explain multidrug resistance and environmental persistence in *C. auris*, especially those involving efflux systems, cell-wall remodeling, and stress-response pathways [4,23,29]. Addressing these gaps will require multidisciplinary approaches integrating genomic, transcriptomic, and proteomic data. Such strategies could help reconstruct regulatory networks governing disinfectant tolerance and biofilm formation [19,23]. In parallel, the integration of artificial intelligence–driven diagnostics with real-time genomic surveillance platforms offers strong potential to enhance early detection and outbreak prediction, particularly in resource-limited settings [17,21].

For *C. glabrata*, both intrinsic and acquired antifungal tolerance continue to complicate clinical management [12,34]. Future research should prioritize the identification of novel therapeutic targets within metabolic and stress-response pathways, including the high-osmolarity glycerol (HOG) and target of rapamycin (TOR) signaling systems [14,38]. In addition, combination strategies integrating existing antifungal agents with adjunctive compounds, such as efflux pump inhibitors, warrant systematic evaluation to overcome resistance [31,35]. Further investigation into within-host microevolution, particularly the temporal dynamics of resistance emergence under antifungal pressure, will be essential for understanding treatment failure and optimizing therapeutic strategies [13,15].

Comparative studies integrating molecular, phenotypic, and epidemiological data from *C. auris* and *C. glabrata* are also needed to define shared and divergent mechanisms of virulence, stress adaptation, and resistance evolution [4,12]. Such approaches could clarify evolutionary processes, including clade replacement in *C. auris* and sequence type–driven diversification in *C. glabrata*, that contribute to the emergence and spread of antifungal resistance [13,21,22].

The development of rapid, scalable, and cost-effective diagnostic tools remains a critical priority. Expanding access to such technologies in low- and middle-income countries (LMICs) is essential to avoid underestimation of the global burden of antifungal-resistant *Candida* infections [1,3]. Strengthening local diagnostic capacity, including training healthcare professionals and implementing point-of-care testing within decentralized healthcare systems, will be key to enabling timely detection and intervention [17,18].

Finally, effective infection control and environmental decontamination strategies remain central to limiting the spread of *C. auris* [7,8,26]. The incorporation of real-time genomic tracking into infection-control programs will facilitate early identification of emerging clades and resistant lineages [10,25]. More broadly, progress in managing these pathogens will depend on coordinated international data sharing and collaborative translational research involving clinicians, microbiologists, epidemiologists, public health policymakers, and industry partners [1,31]. Establishing standardized global databases for antifungal resistance and clade distribution will further strengthen cross-border surveillance and support the development of region-specific clinical guidelines [3,5].

Collectively, future progress will depend on a transition toward integrated, data-driven, and precision-based strategies that combine molecular insight, real-time surveillance, and targeted therapeutic interventions to effectively counter the evolving threat of antifungal resistance [1,3].

## 7. Conclusions and Outlook

*C. auris* and *C. glabrata* remain among the most serious fungal threats to global healthcare because of their critical levels of multidrug resistance, remarkable adaptability, and capacity to colonize healthcare settings. Their epidemiology, genetic divergence, and antifungal resistance systems continue to be actively characterized. However, growing evidence suggests that current knowledge remains insufficient to fully control and manage these pathogens effectively. Addressing the molecular mechanisms underlying multidrug tolerance, environmental persistence, and biofilm formation therefore remains a central priority.

Importantly, *C. auris* and *C. glabrata* exhibit distinct evolutionary patterns of antifungal resistance. In *C. auris*, resistance is primarily driven by clade expansion and replacement of highly adapted lineages, whereas in *C. glabrata*, resistance predominantly emerges through sequence type-associated diversification and within-host microevolution under antifungal selective pressure. These findings suggest that antifungal resistance in *Candida* species is mediated by pathogen-specific evolutionary mechanisms rather than a single universal pathway. A deeper understanding of these mechanisms will facilitate the identification of novel therapeutic targets and support the development of more effective antifungal strategies.

Although species identification has improved through biochemical methods and mass spectrometry, including MALDI-TOF, limited accessibility in resource-limited settings highlights the urgent need for rapid, cost-effective, and user-friendly point-of-care diagnostic tools to enable timely therapeutic intervention. Furthermore, reliable estimates of the global burden of antifungal-resistant *Candida* infections, particularly in low- and middle-income countries (LMICs), remain difficult to obtain. Insufficient diagnostic infrastructure and limited surveillance capacity are likely to contribute to significant underestimation of disease burden.

The continued rise in multidrug-resistant fungal pathogens, particularly strains resistant to triazoles and echinocandins, could further restrict available treatment options. However, the recent development of newer antifungal agents, including rezafungin and ibrexafungerp, provides cautious optimism. Rezafungin has demonstrated promising in vitro and in vivo activity against *C. auris*, including echinocandin-resistant isolates [43,44]. However, further investigation is warranted to determine its clinical efficacy against echinocandin-resistant *C. glabrata* bloodstream infections. Well-designed clinical studies are needed to evaluate its efficacy, safety, and applicability across diverse patient populations and healthcare settings.

As *C. auris* continues to spread globally, integrated strategies that elucidate its molecular mechanisms, optimize combination therapies, strengthen infection control and environmental decontamination measures, and expand global surveillance systems are essential to limit further dissemination. Real-time genomic surveillance integrated within international health networks will be critical for early detection of emerging clades and resistant lineages. In contrast, effective management of *C. glabrata* requires greater emphasis on antifungal stewardship, early detection of resistance development and monitoring of host evolutionary dynamics during therapy.

Taken together, these findings suggest that *C. auris* and *C. glabrata* should not be managed within a single unified framework despite their shared multidrug-resistant phenotypes. Rather, their distinct evolutionary strategies, i.e., clonal transmission in *C. auris* versus therapy-driven microevolution in *C. glabrata*, necessitate pathogen-specific approaches to surveillance, diagnosis, and treatment. In the absence of such tailored strategies, continued reliance on generalized antifungal management could accelerate resistance development, compromise therapeutic efficacy, and hinder long-term advances in antifungal drug development and infection control.

Ultimately, integrating molecular insights with real-time clinical and epidemiological data will be essential to transition from reactive management to predictive, precision-based antifungal strategies. Addressing these gaps will be critical for improving global preparedness, strengthening healthcare system resilience, and reducing the clinical burden of antifungal-resistant *Candida* infections.

## Figures and Tables

**Table 2:** Molecular mechanisms and clinical impact of antifungal resistance in *C*. *glabrata*.

Drug Class/Resistance Type	Molecular Mechanism/Genes Involved	Effect	Clinical Impact	References
Azole resistance	Gain-of-function mutations in *PDR1*; overexpression of efflux pumps (*CgCDR1, CgCDR2, PDH1, SNQ2*)	Reduced intracellular azole accumulation via efflux activity	Major driver of multidrug resistance; limits azole efficacy in candidemia	[12,35]
Indirect azole resistance	Mutations in *CgHXT4/6/7* (hexose transporters regulating azole uptake)	Reduced azole import; resistance even in *PDR1* wild-type strains	Harder to predict; bypasses standard molecular markers	[13]
Ergosterol regulation (azole-related)	Deletion of *HAP1B* contributes to reduced *ERG* gene expression under hypoxic stress	Increased azole susceptibility; links oxygen stress to ergosterol regulation	Highlights hypoxia as a modulator of antifungal response; potential therapeutic target	[38]
Echinocandin resistance	Point mutations in *FKS1* and *FKS2* (hotspot 1 and 2 regions)	Reduced β-1,3-glucan synthase activity; decreased susceptibility to echinocandins	Treatment failure in bloodstream infections; requires early detection	[34,39]
Emerging echinocandin resistance	Gene conversion between *FKS1* and *FKS2*, forming hybrid alleles	Reduced echinocandin binding affinity; complicates molecular diagnostics	Hidden resistance under therapeutic pressure; challenges detection	[15]
Cell wall remodeling	Mutations in *CHS3*, *CHS3B*, KRE5; increased chitin deposition	Reinforced cell walls; resistance to micafungin and detergents (e.g., SDS)	Cross-resistance to environmental stressors; potential hospital outbreak marker	[36,42]
Biofilm-associated resistance	Upregulation of *EPA1*, *EPA6*, *EPA7*; biofilm regulators (*BCR1, ACE2, YAK1*)	Poor drug penetration, metabolic quiescence, and stress adaptation	Major factor in catheter/mucosal infections; tolerance persists during therapy	[35,41]
Drug-tolerant persisters	Non-proliferative macrophage-resident cells; oxidative stress–adapted and tolerant to echinocandins	Serve as reservoirs for resistance; only amphotericin B can eradicate persisters	Hidden reservoirs of recurrence; novel target for antifungal development	[40]

**Table 3:** Comparative characteristics of *Candida auris* and *Candida glabrata*.

Feature	*Candida auris*	*Candida glabrata*
First identification	2009 (Japan)	1995 (reclassified from *Saccharomyces*)
Epidemiology	Emerging global pathogen; hospital outbreaks; clade-specific distribution	Endogenous opportunistic pathogen; widely distributed; increasing incidence
Transmission	High nosocomial transmission; environmental persistence	Limited person-to-person transmission; mainly endogenous
Drug resistance profile	Frequently multidrug-resistant (azole, amphotericin B, echinocandin)	Intrinsically reduced azole susceptibility; acquired echinocandin resistance
Genetic diversity	Distinct clades (I–VI) with geographic segregation	High genetic plasticity; sequence types and microevolution
Biofilm formation	Moderate; clade-dependent	Variable but clinically significant biofilm formation
Environmental survival	High (survives on surfaces, disinfectants)	Moderate
Virulence	Variable; often lower than *C. albicans* but outbreak-prone	Lower virulence but persistent infections
Diagnostic challenges	Misidentification common without MALDI-TOF/WGS	Easier identification; sufficient standard methods
Clinical impact	Severe outbreaks; high mortality in ICU settings	Persistent candidemia; recurrent infections

## Data Availability

No new data were created or analyzed in this study. Data sharing is not applicable to this article.
